# Cases of Meningococcal Disease Associated with Travel to Saudi Arabia for Umrah Pilgrimage — United States, United Kingdom, and France, 2024

**DOI:** 10.15585/mmwr.mm7322e1

**Published:** 2024-06-06

**Authors:** Madhura S. Vachon, Anne-Sophie Barret, Jay Lucidarme, John Neatherlin, Amy B. Rubis, Rebecca L. Howie, Shalabh Sharma, Daya Marasini, Basanta Wagle, Page Keating, Mike Antwi, Judy Chen, Tingting Gu-Templin, Pamala Gahr, Jennifer Zipprich, Franny Dorr, Karen Kuguru, Sarah Lee, Umme-Aiman Halai, Brittany Martin, Jeremy Budd, Ziad Memish, Abdullah M. Assiri, Noha H. Farag, Muhamed-Kheir Taha, Ala-Eddine Deghmane, Laura Zanetti, Rémi Lefrançois, Stephen A. Clark, Ray Borrow, Shamez N. Ladhani, Helen Campbell, Mary Ramsay, LeAnne Fox, Lucy A. McNamara

**Affiliations:** ^1 ^Epidemic Intelligence Service, CDC; ^2^Santé publique France, Saint Maurice, France;^ 3^Meningococcal Reference Unit, UK Health Security Agency, Manchester Royal Infirmary, Manchester, United Kingdom; ^4^Division of Bacterial Diseases, National Center for Immunization and Respiratory Diseases, CDC; ^5^ASRT, Inc, Smyrna, Georgia; ^6^New York City Department of Health and Mental Hygiene, New York, New York; ^7^Minnesota Department of Health; ^8^Hennepin County Public Health, Minneapolis, Minnesota; ^9^Los Angeles County Department of Public Health, Los Angeles, California; ^10^California Department of Public Health; ^11^Ohio Department of Health; ^12^College of Medicine, Alfaisal University, Riyadh, Saudi Arabia; ^13^Ministry of Health, Riyadh, Saudi Arabia; ^14^CDC Middle East and North Africa Regional, Office of the Director, Global Health Center; ^15^Invasive Bacterial Infections Unit and the National Reference Center for Meningococci and Haemophilus Influenzae, Institut Pasteur, Paris, France; ^16^Immunisation Division, UK Health Security Agency, Colindale, London, United Kingdom.

**Summary**

**What is already known about this topic?**

Outbreaks of meningococcal disease can occur in conjunction with large gatherings, including Islamic Hajj and Umrah pilgrimages.

**What is added by this report?**

Twelve meningococcal disease cases associated with Umrah travel to Saudi Arabia have been identified. Nine patients were unvaccinated; vaccination status of three patients was unknown. Ciprofloxacin-resistant strains were identified in three of 11 cases with available antimicrobial susceptibility testing data.

**What are the implications for public health practice?**

Pilgrims aged ≥1 year entering Saudi Arabia should have received a quadrivalent meningococcal (MenACWY) vaccine within the last 3–5 years (depending on vaccine type). Rifampin, ceftriaxone, or azithromycin should be preferentially considered for prophylaxis of close contacts of Saudi Arabia travel–associated cases.

Invasive meningococcal disease (IMD), caused by infection with the bacterium *Neisseria meningitidis*, usually manifests as meningitis or septicemia and can be severe and life-threatening (*1*). Six serogroups (A, B, C, W, X, and Y) account for most cases (*2*). *N. meningitidis* is transmitted person-to-person via respiratory droplets and oropharyngeal secretions. Asymptomatic persons can carry *N. meningitidis *and transmit the bacteria to others, potentially causing illness among susceptible persons. Outbreaks can occur in conjunction with large gatherings (*3*,*4*). Vaccines are available to prevent meningococcal disease. Antibiotic prophylaxis for close contacts of infected persons is critical to preventing secondary cases (*2*).

Umrah, an Islamic pilgrimage to Mecca, Saudi Arabia, can be performed at any time during the year. Hajj is an annual Islamic pilgrimage, occurring this year during June 14–19. Hajj and Umrah pilgrimages attract millions of travelers annually from more than 184 countries (*4*). In 2024, 30 million pilgrims performed Umrah during the month of Ramadan (March 10–April 8, 2024); approximately 13.5 million were international travelers (Z Memish, MD, AlFaisal University, personal communication, May 2024).*

Large meningococcal disease outbreaks associated with Hajj and Umrah were reported in 1987, 1992, and 2000–2001 (*4*). Since 2002, Saudi Arabia has required documentation of either a quadrivalent meningococcal (MenACWY) polysaccharide vaccine within the last 3 years or a MenACWY conjugate vaccine within the last 5 years and administered ≥10 days before arrival for all pilgrims aged ≥1 year entering the country.^†^ However, enforcing this requirement is challenging, because Umrah can occur at any time of year, and many pilgrims are not traveling on an Umrah-specific visa. One study estimated vaccination compliance for Umrah to be 41% (*4*). Several studies have examined vaccination coverage among Hajj pilgrims, reporting highly variable estimates (*4*). An investigation was initiated after reports in 2024 of Umrah-associated IMD cases in the United States, the United Kingdom, and France.

## Investigation and Outcomes

On April 17, 2024, CDC was notified of two IMD cases^§^ in the United States in persons with recent Umrah travel to Saudi Arabia. On April 23, public health authorities in the United Kingdom and France alerted CDC to additional Umrah travel–associated cases in those countries. CDC issued an Epidemic Information Exchange (Epi-X)^¶^ notice on April 24, requesting that U.S. jurisdictions report any Saudi Arabia travel–associated IMD cases. This activity was reviewed by CDC, deemed not research, and was conducted consistent with applicable federal law and CDC policy.**

As of May 29, 12 Saudi Arabia travel–associated cases have been identified from three countries: the United States (five), France (four), and the United Kingdom (three). Seven patients were male, and five patients were female. Two cases occurred in persons aged 0–12 years, four each among adults aged 25–44 and 45–64 years, and two among adults aged ≥65 years. The 10 adult patients traveled to Saudi Arabia, and the two child patients were household contacts of a nonpatient asymptomatic adult traveler. Nine patients were unvaccinated, and the vaccination status of three patients was unknown. All travelers visited Saudi Arabia during March–May 2024, and symptom onset occurred upon return to their country of origin in April and May (Figure).

**FIGURE:**
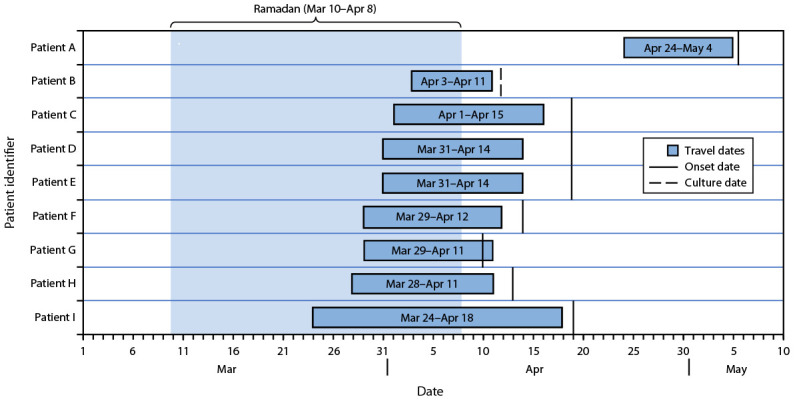
Dates of symptom onset* and Umrah-related travel among nine patients^†^ who had received positive test results for invasive meningococcal disease after travel to Saudi Arabia — United States, United Kingdom, and France, March–May 2024 * Culture date is indicated for one patient for whom reported onset date reflected symptoms unrelated to meningococcal disease. The travel dates for index travelers are shown for cases that occurred among persons who were close contacts of travelers. ^†^ Exact travel and onset dates were unavailable for three patients.

Isolates from 11 patients were available for whole-genome sequencing, 10 of which were identified as *N. meningitidis* serogroup W (NmW, sequence type ST-11, clonal complex CC11), and one (from a U.S. patient) was serogroup C (NmC, ST-12790, CC4821). The U.S. NmC isolate, one U.S. NmW isolate, and one French NmW isolate had a genomic marker (*gyrA* T91I) for ciprofloxacin resistance. Antimicrobial susceptibility testing conducted for nine NmW isolates confirmed that two were resistant to ciprofloxacin. Serogroup and antimicrobial susceptibility could not be determined for one U.S. case because no isolate was available.

## Preliminary Conclusions and Actions

Although vaccination is required for Hajj and Umrah pilgrims, all identified cases occurred among persons who were either unvaccinated or whose vaccination status was unknown. It is important that persons considering travel to perform Hajj or Umrah consult with their health care providers, and providers can ensure that pilgrims aged ≥1 year have received a MenACWY vaccine within the last 3–5 years (depending upon vaccine type received) and ≥10 days before entering Saudi Arabia (*4*). Pilgrims should seek immediate medical attention if they develop signs or symptoms consistent with meningococcal disease.^††^

Health departments should ascertain whether patients with meningococcal disease have traveled to Saudi Arabia or been in close contact with travelers to Saudi Arabia. CDC has published guidance on parameters specifying antibiotic selection for prophylaxis of close contacts of meningococcal disease patients (*5*). Close contacts of people with meningococcal disease should receive antibiotic chemoprophylaxis as soon as possible after exposure, regardless of immunization status, ideally < 24 hours after the index patient is identified. Aligned with this guidance and considering that ciprofloxacin-resistant strains were identified in three of 11 cases with available information, prophylaxis with rifampin, ceftriaxone, or azithromycin should be preferentially considered instead of ciprofloxacin for close contacts of patients with Saudi Arabia travel–associated cases.^§§^

## Footnotes

* https://gulfnews.com/world/gulf/saudi/saudi-arabia-umrah-pilgrims-in-ramadan-topped-30-million-1.1713008730892

† https://www.moh.gov.sa/en/HealthAwareness/Pilgrims_Health/Documents/Health-Regulations-En.pdf; https://www.moh.gov.sa/en/HealthAwareness/Pilgrims_Health/Documents/Health-Regulations-Umrah-EN.pdf

§
https://ndc.services.cdc.gov/case-definitions/meningococcal-disease-2015/

¶
https://www.emergency.cdc.gov/epix/index.asp

** 45 C.F.R. part 46.102(l)(2), 21 C.F.R. part 56; 42 U.S.C. Sect. 241(d); 5 U.S.C. Sect. 552a; 44 U.S.C. Sect. 3501 et seq.

†† Signs and symptoms might include fever, headache, stiff neck, nausea, vomiting, photophobia or altered mental status (meningitis) or fever, chills, fatigue, vomiting, cold extremities, severe aches and pains, rapid breathing, diarrhea, and, in advanced stages, a petechial or purpuric rash (meningococcemia).

§§
https://emergency.cdc.gov/han/2024/han00508.asp
